# Discrete bisoliton fiber laser

**DOI:** 10.1038/srep34414

**Published:** 2016-10-21

**Authors:** X. M. Liu, X. X. Han, X. K. Yao

**Affiliations:** 1 State Key Laboratory of Modern Optical Instrumentation, Department of Optical Engineering, Zhejiang University, Hangzhou 310027, China; 2 School of Physics and Electronic Science, Hunan University of Science and Technology, Xiangtan 411201, PR China; 3 State Key Laboratory of Transient Optics and Photonics, Xi’an Institute of Optics and Precision Mechanics, Chinese Academy of Sciences, Xi’an 710119, China

## Abstract

Dissipative solitons, which result from the intricate balance between dispersion and nonlinearity as well as gain and loss, are of the fundamental scientific interest and numerous important applications. Here, we report a fiber laser that generates bisoliton – two consecutive dissipative solitons that preserve a fixed separation between them. Deviations from this separation result in its restoration. It is also found that these bisolitons have multiple discrete equilibrium distances with the quantized separations, as is confirmed by the theoretical analysis and the experimental observations. The main feature of our laser is the anomalous dispersion that is increased by an order of magnitude in comparison to previous studies. Then the spectral filtering effect plays a significant role in pulse-shaping. The proposed laser has the potential applications in optical communications and high-resolution optics for coding and transmission of information in higher-level modulation formats.

Solitons, which are the localized formations in nonlinear systems^1^, appear in various physical settings^2^,^3^,^4^,^5^,^6^,^7^,^8^,^9^,^10^,^11^,^12^,^13^,^14^,^15^. The soliton on a water surface was first described by John Scott Russell and the fundamental concept of soliton in mathematical physics was first introduced by Norman Zabusky and Martin Kruskal^10^,^11^. Physically, temporal solitons in conservative systems can be considered as a result of a balance between nonlinearity and dispersion. Dissipative solitons differ from conservative ones in that gain and loss play a significant role in their formation^1^. Lasers are one example of systems generating dissipative solitons^1^. Due to the complex balance, dissipative solitons have normally fixed shape^1^. As a result, energy of a single dissipative soliton is limited. Therefore, higher pump levels lead to generation of multiple solitons^16^,^17^. Generally, lasers admit multi-pulsing^18^,^19^, harmonic mode locking^20^,^21^, and bound states^22^,^23^,^24^,^25^,^26^,^27^,^28^,^29^,^30^,^31^. The bound-state solitons have been experimentally observed in fiber lasers with various mode-locking techniques^23^,^26^,^31^,^32^, including nonlinear polarization rotation, figure-of-eight lasers, and carbon nanotube (CNT) mode-locking. Variety of different soliton characteristics have been obtained^23^,^24^,^25^,^26^,^27^,^31^,^32^,^33^,^34^,^35^,^36^,^37^. Stable bound states of two solitons have potential applications in optical communications for coding and transmission of information in higher-level modulation formats, increasing capacity of communication channels beyond binary coding limits^8^,^30^,^31^,^38^,^39^,^40^.

The fast development in the fiber Bragg grating (FBG) fabrication technology provides an excessive amount of negative dispersion. Namely, a 10-cm-long grating can compensate the dispersion acquired over standard fiber length of 50 km^41^,^42^,^43^. Moreover, FBG is also an ideal filter and wavelength selection component for ultrafast broadband fiber lasers. Thus, a laser cavity with large anomalous dispersion and narrow filter bandwidth would strongly influence the composite balance within the dissipative solitons creating their new forms.

Bisolitons and multisolitons had been investigated by Stratmann *et al.*^38^ and the stable multisoliton solutions were studied by Akhmediev *et al.* by means of energy and momentum balance equations^44^. Malomed had proposed a general method to find an effective potential of interaction between far separated solitons^45^. The interaction between weakly overlapping pulses in quintic Ginzburg-Landau equation and the driven damped NLSE was described by Afansajev *et al.*^46^. These techniques allow us to predict and observe quantized separations between the soitons in a pair^47^. In this paper, we made further theoretical, numerical and experimental studies of bisolitons in fiber lasers that have multiple discrete equilibrium distances with the quantized separations. We also present the experimental results on the bisolitons with multiple discrete equilibrium distances, which are delivered from a largely anomalous dispersion FBG-based fiber laser with a narrow filter. The novel feature of our FBG-based laser is the total dispersion of the cavity that is up to −10 ps^2^, which is more than an order-of-magnitude increase in comparison to the previous studies, and the FBG-based filter is as narrow as ~0.9 nm.

## Results

### Experiment setup

**Figure 1:**
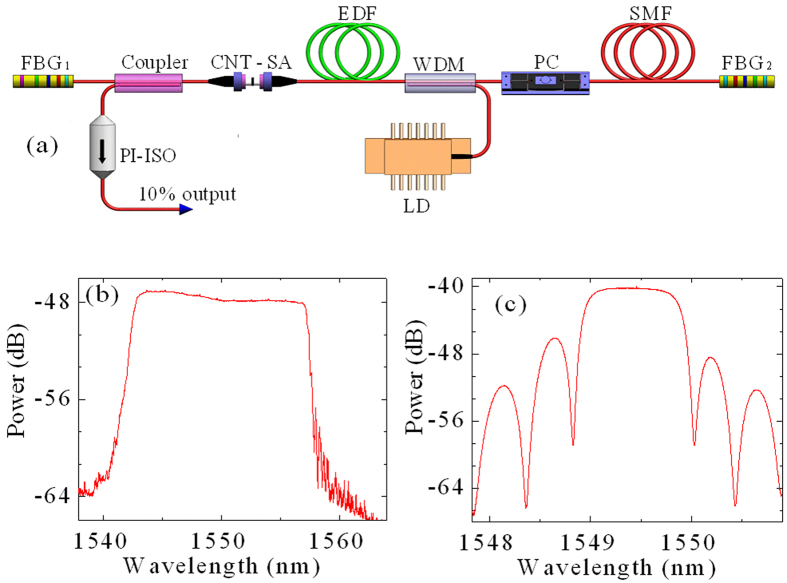
(**a**) Laser setup. EDF, erbium-doped fiber; SMF, single-mode fiber; WDM, wavelength-division multiplexer; PC, polarization controller; LD, laser diode; FBG, fiber Bragg grating; CNT-SA, carbon nanotube saturable absorber; PI-ISO, polarization independent isolator. Reflection spectra of (**b**) FBG_1_ and (**c**) FBG_2_.

The schematic diagram of large anomalous dispersion fiber laser is shown in Fig. 1(a). The laser system consists of two FBGs, a fused coupler with 10% output ratio, a CNT saturable absorber (SA), a 5-m-long erbium-doped fiber (EDF) with 6 dB/m absorption at 980 nm, a wavelength-division multiplexer (WDM), a polarization controller (PC), and a single-mode fiber (SMF). The EDF and SMF have dispersion parameters of about 11.6 and −22 ps^2^/km at 1550 nm, respectively. FBG_1,2_ have the super-Gaussian reflection profile with the 3-dB bandwidth of ~15 nm (Fig. 1(b)) and ~0.9 nm (Fig. 1(c)). FBG_2_ serves as the narrow filter and induces the spectral filtering effect on the soliton evolution. The dispersion of FBG_1_ is about −5 ps^2^/cm with the length of ~15 mm and the central transmittance wavelength of 1559.5 nm. The central wavelength of FBG_2_ is tunable with the dispersion of about −2.5 ps^2^/cm and the length of ~10 mm. The total dispersion of laser cavity is about −10 ps^2^ and the total cavity length is ~43 m. The integrated CNT-based fiber device is realized by sandwiching a ~2 mm^2^ sample between two fiber connectors, as shown in our previous report^48^.

### Theoretical modeling

We numerically simulated the pulse formation and evolution in the laser cavity with circulating pulse. The modeling includes the Kerr effect, the group velocity dispersion of fiber, the dispersion of FBGs, the saturable absorption of CNT, and the saturated gain with a finite bandwidth. In the lumped model, we follow the circulation of the optical pulses in the laser cavity and consider every action of the cavity components on the pulses. When the pulses encounter cavity components, we take into account their effect by multiplying the optical field by the transfer matrix of a particular component.

When the optical pulses propagate through the fiber, the extended nonlinear Schrödinger equation is used to simulate the dynamics and evolution of the pulses, i.e.^48^


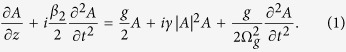


Here *A*, *β*_2_, and *γ* represent the electric filed envelop of the pulse, the fiber dispersion, and the cubic refractive nonlinearity of the fiber, respectively. The variables *t* and *z* are the time and the propagation distance, respectively. When the pulses propagate along the SMF, the first and last terms on the right-hand side of Eq. (1) are ignored. Ω_*g*_ denotes the bandwidth of the gain spectrum. *g* describes the gain function for the EDF and is given by^48^,^49^


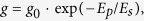


where *g*_0_, *E*_*p*_, and *E*_*s*_ are the small-signal gain coefficient related to the doping concentration, the pulse energy, and gain saturation energy that relies on pump power, respectively.

The normalized absorption of CNT-SA in Fig. 1(a) are fitted according to a simple two-level saturable absorber model^48^,^50^


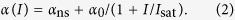


Here *α*(*I*) is the intensity-dependent absorption coefficient, and *α*_0_, *α*_ns_ and *I*_sat_ are the linear limit of saturable absorption, nonsaturable absorption, and saturation intensity, respectively.

When the pulse propagates through FBG in Fig. 1(a), an additional phase is imposed, which can be expressed by^51^


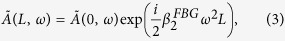


where *ω* is the angular frequency, 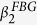 is the second-order dispersion of FBG, and *L* is the length of FBG. FBG_2_ not only imposes the phase on the pulse, but also induces the spectral filtering effect. The reason is that the spectral bandwidth of FBG_2_ is ~0.9 nm, which is much narrower than the bandwidth of FBG_1_ and the gain bandwidth.

In the simulations, the calculation starts with an arbitrary electric field *A*. After one round-trip circulation in the cavity, the obtained results are used as the input of the next round of calculation until the optical field converges to a fixed pulse profile. To match the experimental conditions, we use the following parameters: *g*_0_ = 6 dB/m, Ω_g_ = 25 nm, *E*_*s*_ = 55 pJ, *γ = *4.5 W^−1^km^−1^ for EDF, *γ = *1.3 W^−1^km^−1^ for SMF. The parameters for CNT-SA are set with the values measured^48^, i.e. *α*_0_ = 12.05%, *α*_ns_ = 87.87%, and *I*_sat_ = 9.67 MW/cm^2^. Equation (1) is solved with a predictor–corrector split-step Fourier method^52^.

### Theoretical analysis

The equilibrium distances can be found in various ways. A simple qualitative analysis is the method of effective potential created by the interaction of the tails of individual solitons^22^,^53^. The tails are assumed rigid for the method to give reliable results. According to the full Hamiltonian, the soliton-soliton interaction is given by


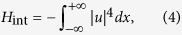


where 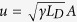 and 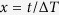. *L*_*D*_ is the dispersion length and 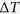 is the pulse duration. By means of the linear superposition of the overlapping solitons (i.e., 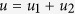), the effective potential *U* of the soliton-soliton interaction can be approximated by^22^,^53^


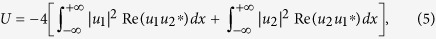


where *u*_1_ and *u*_2_ are two soliton solutions of Eq. (1), which can be expressed as


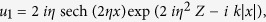



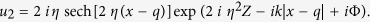


Here 
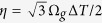
, 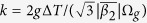, 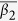 is the average dispersion of laser cavity, *q* is the normalized separation of bisolitons (i.e., *q* = *τ*/Δ*T*), *τ* is the pulse separation of bisolitons, *Z* is the normalized propagation distance, and 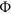 is the phase difference of pulse molecules. Substituting the definition of the variables of 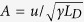 and 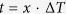 into Eq. (1) and after some manipulation, Eq. (5) can be simplified by


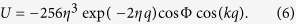


From Eq. (6), the interaction force of the two solitons is


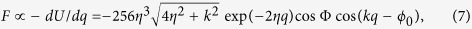


where 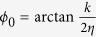.

At the local minima of the effective potential *U*, the bisolitons are at the stationary state. To find the local minima of *U*, the first-order and second-order derivatives of *U* should be zero and more than zero^54^, respectively. That is *dU/dq* = 0 and *dU*^2^*/dq*^2^ > 0. After some manipulation, the equilibrium distance *τ*_*n*_ for the in-phase SMs (i.e., 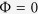) is given by


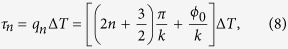


where *n* is the positive integer. From Eq. (8), it is straightforward to see that bisolitons can be formed at the discrete equilibrium distances of *τ*_*n*_. As the soliton tails are not necessarily rigid, the above result can be considered as a simple qualitative approach sufficient for our purposes. One of the deviations from these results is the phase difference between the solitons that can be different form 0 or π^55^.

### Simulation results

**Figure 2:**
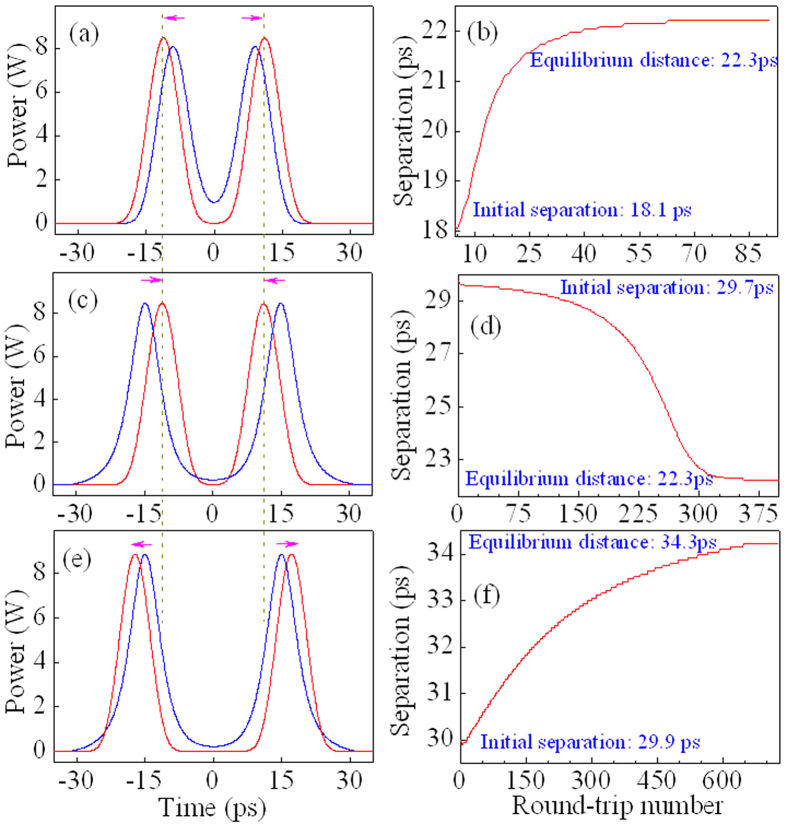
Evolution of in-phase bisolitons on the numerical simulations. Pulse profiles at the initial separation and the equilibrium distance of (**a**) 18.1 and 22.3 ps, (**c**) 29.7 and 22.3 ps, and (**e**) 29.9 and 34.3 ps, respectively. (**b**,**d**,**f**) Evolution of pulse separation of bisolitons with respect to the round-trip number for (**a**,**c**,**e**), respectively. Whether bisolitons are launched with narrower (top) or wider (center) separation, they relax towards their equilibrium distance (dashed lines). There are two different equilibrium distances (center, bottom) although bisolitons are launched with the approximately equal separation. The Supplementary Materials demonstrate the evolution of bisolitons from the initial separation to the equilibrium distance in detail.

The numerical simulations show that two solitons repel from narrower separation, whereas they attract from wider separation; in either case they return to the equilibrium distance. Figure 2(a,c) demonstrate that two solitons repel or attract when their initial separation is 18.1 ps or 29.7 ps, respectively. Finally, they evolve to the equilibrium distance of 22.3 ps. Figure 2(b,d) show the evolution of pulse separation of bisolitons from 18.1 ps to 22.3 ps and from 29.7 to 22.3 ps, respectively. However, two solitons repel from the initial separation of 29.9 ps (Fig. 2(e)), rather than attract from the initial separation of 29.7 ps (Fig. 2(c)), although their initial separations are close to each other. Then, the equilibrium distance is 34.3 ps (Fig. 2(f)) rather than 22.3 ps (Fig. 2(d)). The numerical results show that there are multiple equilibrium distances (e.g.,~46.3 and ~58.3 ps) besides 22.3 and 34.3 ps. The evolution of bisolitons from the initial separation (18.1, 29.7, and 29.9 ps) to the equilibrium distance (22.3 and 34.3 ps) is demonstrated in the Supplementary Materials in detail.

**Figure 3:**
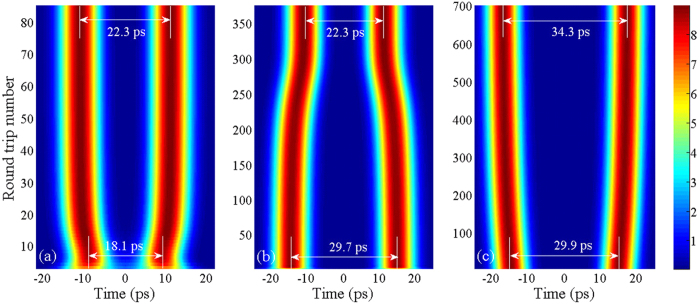
Dynamic evolutions of bisolitons from initial separation to equilibrium distance. (**a**) From 18.1 to 22.3 ps, (**b**) from 29.7 to 22.3 ps, and (**c**) from 29.9 to 34.3 ps.

Figure 3 shows three examples for the dynamic evolutions of bisolitons from the initial separation to the equilibrium distance. The dynamic evolutions from 18.1 to 22.3 ps, from 29.7 to 22.3 ps, and from 29.9 to 34.3 ps are demonstrated in Fig. 3(a–c), respectively. Figure 3(a) shows that two solitons repel from narrower initial separation of 18.1ps, whereas they attract from wider initial separation of 29.7 ps (Fig. 3(b)). After ~90 or ~400 of round-trip number, they return to the same equilibrium distance of 22.3 ps. Figure 3(c) illustrates that two solitons evolve to another equilibrium distance of 34.3 ps when the initial separation is 29.9 ps rather than 29.7 ps.

**Figure 4:**
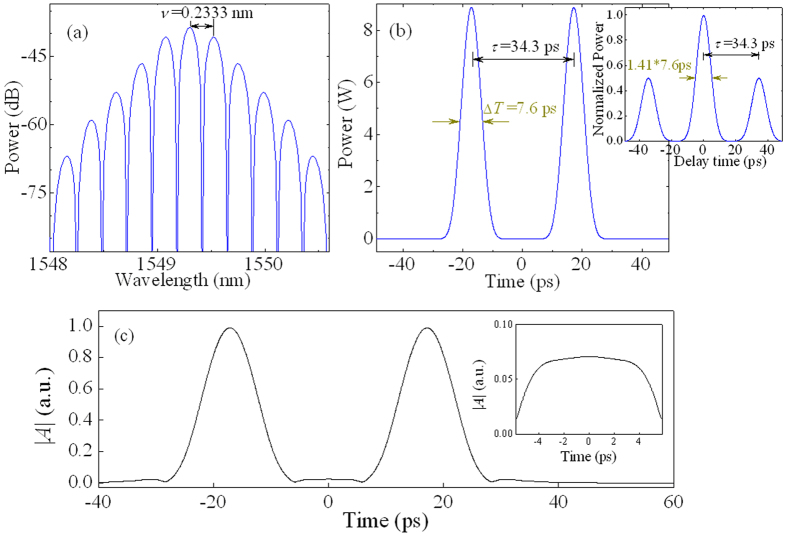
Typically numerical results for in-phase bisolitons. (**a**) Optical spectra. (**b**) Pulse profiles and the corresponding autocorrelation trace (inset). (**c**) Absolute value of sum for two optical fields, where the inset is the enlarged image of overlapped optical field region. The modulation period *ν* of optical spectrum is 0.2333 nm. The pulse duration Δ*T* of bisolitons is 7.6 ps with the separation *τ* of 34.3 ps.

The typical results of numerical simulations for in-phase bisolitons are shown in Fig. 4. It is seen from Fig. 4 that the optical spectrum is modulated with the period *ν* of 0.2333 nm and the pulse separation *τ* of bisolitons is 34.3 ps with the pulse duration Δ*T* of 7.6 ps. Note that the pulse separation of 34.3 ps is an equilibrium distance of bisolitons. The inset in Fig. 4(b) is the autocorrelation trace of bisolitons. We can see that the optical fields of two identical solitons with the separation of 34.3 ps are overlapped in Fig. 4(c). The inset of Fig. 4(c) shows the zoomed-in region of the overlapped area.

### Experiments and comparisons

**Figure 5:**
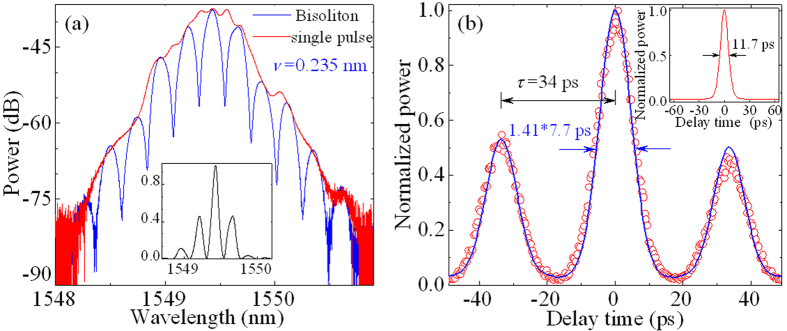
Typical experimental results. (**a**) Optical spectra of the single soliton (red curve) and bisoliton (blue curve) operation. The black curve in the inset is the linear scale of bisolitons, corresponding to the blue curve. (**b**) Autocorrelation traces for the single soliton (red curve in inset) and bisoliton (blue curve). The blue curve and red circles are the traces with average times of 16 and single times, respectively.

The proposed laser emits continuous wave (CW) at the pump power of *P* ≈ 10 mW. The self-starting mode-locking is observed at *P* ≈ 30 mW when the polarization controller is appropriately adjusted. The typical experimental results on in-phase bisolitons are shown in Fig. 5(a,b) for the optical spectra and the autocorrelation traces, respectively. The blue and red curves in Fig. 5(a) are the bisoliton and single soliton operations, respectively. The curves in Fig. 5(b) and its inset are the autocorrelation traces for the bisoliton and the single soliton, respectively. The blue curve and red circle in Fig. 5(b) are the trace with average times of 16 and single times, respectively. Two red curves in Fig. 5 show that the single pulse has the 3-dB spectral width of ~0.5 nm and the pulse duration of ~8.3 ps (corresponding to 11.7 ps of the full width at half maximum (FWHM)). We can see from Fig. 5 that the optical spectrum is modulated with the period *ν* of ~0.235 nm and the depth of >15 dB, and the pulse separation *τ* of bisolitons is ~34 ps with the pulse duration Δ*T* of ~7.7 ps. Obviously, the experimental results exhibit that *ν* approximately is equal to the reciprocal of *τ* (i.e., *ν*  ≈ 1/*τ*). The experimental observations (e.g., Fig. 5(b)) are in good agreement with the numerical results (e.g., inset of Fig. 4(b)).

**Figure 6:**
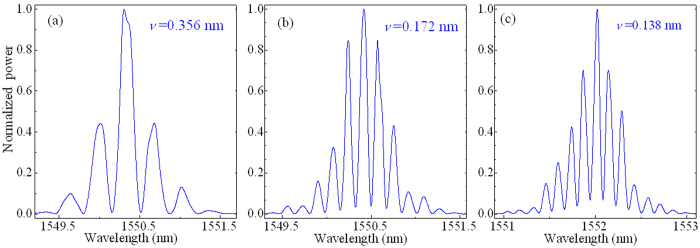
Experimental results of in-phase bisolitons. Optical spectra with the modulation period *ν* of (**a**) ~0.356, (**b**) ~0.172, and (**c**) ~0.138 nm. The corresponding pulse separations *τ* of bisolitons are ~22.5, ~46.5, and ~58.2 ps, respectively.

Figure 6(a–c) are the optical spectra of in-phase bisolitons in the experimental observations, which are modulated with the period of ~0.356, ~0.172, and ~0.138 nm, respectively. According to the theoretical expression of *τ* = 1/*ν*^8^, the pulse separations *τ* of bisolitons are ~22.5, ~46.5, and ~58.2 ps for Fig. 6(a–c), respectively. The experimental observations show that the pulse separation *τ* of bisolitons has some fixed and discrete values, i.e., *τ* is the equilibrium distance in the laser system.

## Discussion

The numerical simulations show that the bisolitons converge towards the equilibrium distance and the experimental observations demonstrate that they have multiple discrete equilibrium distances. To understand the experimental and numerical results well, we provide the qualitative analysis of the interaction of two solitons. The interaction force *F* between the two solitons is periodic but exponentially decreases with the pulse separation due to the exponentially decaying tails. When the separation is slightly narrower than the equilibrium distance *τ*_*n*_, the two solitons repel each other until they return to the balanced position *τ*_*n*_. The solitons will attract one another to *τ*_*n*_ if the separation between them is slightly larger than *τ*_*n*_. Therefore, the bisolitons have the ability to adjust their positions to maintain the balance by the interaction force of repelling or attracting each other. The equilibrium distances *τ*_*n*_ in Eq. (8) are fixed and discrete. This is confirmed by the experimental observations and numerically simulations. In fact, it appears from the experimental and theoretical data that the separation quantization is around 12 ps.

The spectral filtering effect plays an important role in pulse-shaping^56^. The narrow bandwidth Ω_*f*_ of FBG_2_ induces the strong spectral filtering effect, which determines the width of dissipative solitons and the minima of interaction potential^22^,^44^,^57^,^58^. It is obviously that spectral band narrowing widens a pulse due to Δ*T* ∝ 1/Ω_*f*_ 59. At the same time, the narrow filter can enhance the soliton binding because of *E*: 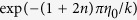 and 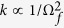 22. A large dispersion is required for keeping a reasonable inter-soliton distance and, simultaneously, it has to be reasonable large for a regime stabilization since the stability parameter 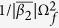 has to be below a critical value for soliton stabilization^59^. Thus, the unique features of our laser setup become directly and obviously connected with the observed phenomena and provide the enhanced stability of the bounded soliton regime^60^,^61^,^62^,^63^.

In conclusion, we have designed a discrete bisoliton fiber laser, which has a spectral filtering with the large anomalous dispersion. The experimental results on the bisolitons with multiple discrete equilibrium distances are reported, which are delivered from a FBG-based fiber laser with a narrow filter. The total dispersion of the proposed laser is up to −10 ps^2^, which is more than an order-of-magnitude increase in comparison with previous lasers, and the FBG-based filter is as narrow as ~0.9 nm, which is much narrower than the gain bandwidth. The dynamics and evolution of the bisoliton fiber laser are investigated experimentally and theoretically. Two solitons in this laser have the ability of restoring their equilibrium separation (Figs 2 and 3), which originates from the balance of repulsive and attractive forces between solitons. Bisolitons here have multiple discrete equilibrium distances with the quantized separations, as confirmed by the theoretical analysis (Eq. (8)) and the experimental observations (Figs 5 and 6). Stable bisoliton bound state presented here has potential applications in optical communications and high-resolution optics for coding and transmission of information in higher-level modulation formats, increasing capacity of communication channels beyond binary coding limits. The proposed laser provides a simple, stable, low-cost ultrafast-pulsed source for spectroscopy, biomedical research and telecommunications.

## Methods

### Measurement method

An optical spectrum analyzer (Yokogawa AQ-6370), an autocorrelator, a 6-GHz oscilloscope, a radio-frequency (RF) analyzer, and a 10-GHz photodetector are used to measure the laser output performances.

## Additional Information

**How to cite this article**: Liu, X. M. *et al.* Discrete bisoliton fiber laser. *Sci. Rep.* **6**, 34414; doi: 10.1038/srep34414 (2016).

## Supplementary Material

Supplementary Movie 1

Supplementary Movie 2

Supplementary Movie 3

Supplementary Information
