# Supplementary material for: Discrete bisoliton fiber laser
Source: Sci Rep. 2016 Oct 21;6:34414. doi: 10.1038/srep34414 (PMC5073350; doi:10.1038/srep34414)
Supplement: Supplementary Information [file srep34414-s4.doc]

Supplemental Information For Manuscript Titled:” **Discrete bisoliton fiber laser**” X. M. Liu^1,2,3^*, X. X. Han^3^, X. K. Yao,^3^ and N. Akhmediev^4^

**X. M. Liu^1,2,3^*, X. X. Han^3^, X. K. Yao,^3^ and N. Akhmediev^4^**

^1^*State Key Laboratory of Modern Optical Instrumentation, Department of Optical Engineering, Zhejiang University, Hangzhou 310027, China*

^2^School of Physics and Electronic Science, Hunan University of Science and Technology, Xiangtan 411201, PR China

^3^State Key Laboratory of Transient Optics and Photonics, Xi’an Institute of Optics and Precision Mechanics, Chinese Academy of Sciences, Xi’an 710119, China

^4^Optical Sciences Group, Research School of Physics and Engineering, Institute of Advanced Studies, The Australian National University, Canberra ACT 0200, Australia

Three videos show the evolution of pulse separation of bisolitons with respect to the round-trip number. movie_fig2_a_ and movie_fig2_c_ show the evolution of pulse separation of bisolitons from 18.1 ps to 22.3 ps(Fig. 2(a)), and from 29.7 to 22.3 ps(Fig. 2(c)), respectively. However, movie_fig2_e_shows thattwo solitons repel from the initial separation of 29.9 ps, rather than attract from the initial separation of 29.7 ps, although their initial separations are close to each other. The final equilibrium distance is 34.3 ps rather than 22.3 ps(Fig. 2(e)).
